# Manipulating objects during learning shrinks the global scale of spatial representations in memory: a virtual reality study

**DOI:** 10.1038/s41598-024-53239-1

**Published:** 2024-02-01

**Authors:** S. Lhuillier, L. Dutriaux, S. Nicolas, V. Gyselinck

**Affiliations:** 1 LaPEA, Université Gustave Eiffel, Université de Paris, 78000 Versailles, France; 2 Laboratoire d’Étude des Mécanismes Cognitifs, Université Lumière Lyon 2, Lyon, France; 3 LMC2, Université Paris-Cité, 92012 Boulogne-Billancourt, France

**Keywords:** Human behaviour, Perception

## Abstract

Goal-directed approaches to perception usually consider that distance perception is shaped by the body and its potential for interaction. Although this phenomenon has been extensively investigated in the field of perception, little is known about the effect of motor interactions on memory, and how they shape the global representation of large-scale spaces. To investigate this question, we designed an immersive virtual reality environment in which participants had to learn the positions of several items. Half of the participants had to physically (but virtually) grab the items with their hand and drop them at specified locations (active condition). The other half of the participants were simply shown the items which appeared at the specified position without interacting with them (passive condition). Half of the items used during learning were images of manipulable objects, and the other half were non manipulable objects. Participants were subsequently asked to draw a map of the virtual environment from memory, and to position all the items in it. Results show that active participants recalled the global shape of the spatial layout less precisely, and made more absolute distance errors than passive participants. Moreover, global scaling compression bias was higher for active participants than for passive participants. Interestingly, manipulable items showed a greater compression bias compared to non-manipulable items, yet they had no effect on correlation scores and absolute non-directional distance errors. These results are discussed according to grounded approaches of spatial cognition, emphasizing motor simulation as a possible mechanism for position retrieval from memory.

## Introduction

### Goal-directed perception and cognition

According to ecological approaches to perception, the world is perceived as a collection of action possibilities^1^. This goal-directed view has influenced many aspects of grounded theories of cognition, which assume that cognition is understood in interaction with environmental contexts^2^, embodied in bodily states^3^ and situated actions^4^. Some grounded conceptions postulate that concepts are also encoded from modal experience in long-term memory in the form of perceptual symbols^5^ that can be retrieved using simulation^6^. Barsalou^7^ described simulation as “the reenactment of perceptual, motor, and introspective states acquired during experience with the world, body and mind”. The discovery of mirror neurons during action observation^8,9^ and the study of mental imagery processes (see^10^ for a review) have provided strong empirical evidence for the existence of simulation processes. Focusing on memory, it has been demonstrated that action-related concepts automatically produce an analogous activity of the corresponding actions in the motor cortex^11,12^, thus introducing a specific type of simulation commonly referred to as motor simulation.

Motor simulation can be seen as evidence that knowledge is embodied in bodily states and goal-directed actions. This phenomenon has been widely studied using a stimulus–response compatibility paradigm: Glenberg and Kaschak^13^ showed in a simple judgment task that directional actions implied by a sentence could facilitate the actual actions (e.g. “open the drawer” elicits shorter responses when the correct answer is performed by moving the hand towards the body to push a button rather than in the opposite direction). Along the same line, Tucker and Ellis^14^ observed that this compatibility effect could also concern the type of motor responses, as power grip responses are faster for objects that imply the same type of grip, but slower for objects that imply a precision grip (and conversely for objects that imply precision grip responses). Note also that the object features that enable grasping (e.g. a handle) are encoded by a specific population of visuo-motor neurons called "canonical neurons". These neurons are recruited even when an object is simply looked at^15^ and not manipulated. More specifically, it has been shown that graspable objects contained within the proximal space of participants elicit more evoked potentials in the primary motor cortex than those located outside of the reachable space^16^. Taken as a whole, it seems that reading or viewing action-related sentences/objects primes the corresponding motor schemes^17,18^ (but see recent debate about replication^19^). Additionally, body postures can interfere with motor simulation, as shown by Dutriaux and Gyselinck^20^: adopting a constrained posture (hands crossed behind one's back) while learning a list of objects negatively impacts retrieval performances for manipulable objects only (compared to non-manipulable objects), whether the objects are presented as images or words. This interfering effect of posture seems to depend on the presence of an action context, as the memory for sentences that contain attentional verbs along with manipulable objects (e.g. “look at a cup”) is not affected by the interfering posture whereas it is affected by action verbs^21^.

These results are consistent with the idea that not only is perception goal-directed^1^, but that representations in memory aim to support action^22^ by using motor simulation as part of the retrieval process^23^. The present work will focus on a specific type of representations about which the specific contributions of motor simulation processes are still to be explored, namely spatial representations retrieved from memory.

### Embodied spatial cognition: different spaces, different actions

Research in spatial cognition is interested in how individuals apprehend and build knowledge about the space that surrounds them. As spatial knowledge is mostly derived from a variety of sensory, action-related and locomotor experiences, spatial context can be considered as a “venue for action”^4,24^. This fits perfectly with grounded theories of cognition, where spatial representations can be described as shaped by the body and its potential for action in relation with the environment (see^25–27^). Yet, it is not clear how actions and motor simulation participate in the retrieval of all spatial representations. In particular, it has been hypothesized that the value of action in spatial memory depends on the scale of the space considered. Several taxonomies of spaces emphasize the functional differences between peripersonal and larger-scale spaces^27–29^.

This dichotomy seems quite intuitive and mainly revolves around the degree of immediate motor interactions between the body and the environment (for reviews see^30,31^). Peripersonal space is *the space around the body*^27,32^ where body specific perception linked action possibilities take place. The evidence of action-directed perceptual processing of peripersonal spaces is abundant, and can be found in regard to a specific parameter of spatial cognition, namely distance perception. For example, the distance between the body and an unreachable object is significantly underestimated if participants are provided with a reach-extending tool^33,34^. This type of action ability effect has also been observed with respect to the individual's own body characteristics, with individuals with narrow shoulders perceiving door apertures as larger in width compared to individuals with broad shoulders^35^. More interestingly, Longo and Lourenco^36^ observed a systematic relation between the estimated extent of peripersonal space and arm length, suggesting that arm length could be used as a reference scale for immediate distances. In addition, the distance between the body and a tool is judged closer if the handle is directly reachable, compared to when the handle is oriented in such a way that the person needs to twist their arm to reach it^37^. Converging results thus led Linkenauger et al. to propose that motor simulation is automatically used during spatial perception to anticipate actions, and that the outcome of the simulation can influence perceived distances according to action capabilities^38^. Nonetheless, these results are the subject of much controversy as some authors argue that they are not related to actual perceptual effects but rather to judgment biases such as complying with experimental demands^39^. We advocate that this specific criticism can be addressed by observing action-directed effects linked with motor simulation not during explicit perception, but during the retrieval of distances from spatial representations encoded in memory. To our knowledge, only one recent study has investigated the relationship between motor simulation and spatial memory following this approach. In Ruggiero et al.^40^ participants had to memorize the positions of three objects that were placed directly in front of them, with either arms free or bent behind the back (interfering posture, negatively impacting motor simulation as in^20^). They were then tested on their memory of the presented configuration by estimating which object was the closest or the farthest in terms of distance (spatial judgement), or which object was to the right or left (categorical judgment only). Spatial judgments could be made with reference to the subject's own frame of reference (“egocentric” recall) or to one of the objects presented (“allocentric” recall). The results showed that the interfering posture only negatively affected distance recall in an egocentric frame of reference, and in none of the other conditions. Strikingly, this effect was not observed when the objects were positioned outside of the arm reaching range of the participants (100 cm away). The authors therefore conclude that egocentric recall of spatial coordinates in peripersonal space from memory recruits motor resources (possibly through motor simulation), and that spatial judgments of coordinates located outside the interaction range instead involve mostly visuo-spatial strategies^40,41^.

According to a constructivist view of spatial memory, spatial representations can be described as “thematic overlays of multimedia information” integrated from different perspectives or extents, in a fragmented, dynamic, and multimodal object^26,27^. Based on this definition, one could hypothesize that if motor simulation is involved during peripersonal spatial processing, it will be integrated along with broader scales into the spatial representation. Yet very little is known about the interaction between peripersonal action-directed processes and the global encoding of spatial features for large-scale places. To our knowledge, only a few studies tackled this question. Princeps works showed that a stimulus described as spatially close to a subject is more easily/quickly accessible from memory than a distant stimulus, both during text comprehension with lexical decision^42^, or map learning tasks^43^. More recently, Thomas et al.^44^ have obtained promising results suggesting that physical interaction with objects leads to remembering object positions as being closer to each other, as well as compressing the external environmental boundaries. This effect does not seem to be restricted to manipulated items since it also affects the memorized distances between more distant items that were not interacted with^45^. Although these studies suggest that spatial representations undergo global metric compression in response to interaction with objects, they used only simple, short-scale, low-ecological environments. Furthermore, they did not investigate the mechanisms from which this compression from peripersonal to environmental space might originate (e.g. motor simulation).

Therefore, the aim of the present work is to address the following question: is motor simulation linked with peripersonal actions involved in the construction and memory of large-scale spatial representations? To reach this goal, we provide a new methodology to study action-directed spatial memory using immersive virtual reality and highly sensitive geometrical analysis of participants’ retrieval of coordinates on maps (instead of subjective judgments or inter-items distances). Our expected results stem from the idea that priming or performing reaching actions can lead to underestimation of proximal distances^46^, and that such perceptual biases would be integrated into the encoding or the retrieval of metric properties information in memory within large-scale spatial representations^45^. We thus hypothesize that motor simulations of arm-reaching towards objects may induce metric compression of the scale of spatial representations. We will compare spatial learning of objects’ coordinates by contrasting two learning conditions: an “active” condition in which individuals reach and grab then drop items at specific locations, and a “passive” condition in which participants only see items appearing at specific locations without interacting with them. According to the postulate that performing a reaching action influences distance perception^38^, we expect participants in the active condition to significantly underestimate distances compared to passive participants, as all items in all conditions will be encomprised within peripersonal space but only those in the active condition will be actually targeted by a directional reaching movement. In addition to motor activity, we will also observe the influence of manipulable objects (such as tools) and non-manipulable objects on spatial memory. Along with the idea that manipulable objects integrate and prime action possibilities in their semantic representations^20^, we expect them to potentiate scaling compression during spatial retrieval more than non-manipulable objects, this effect being stronger for active participants. In sum, we expect that the metric properties of representations of large-scale environment will be influenced by repeated motor interaction experiences or simulations due to the integration of action capability information within the representation, and impacts its memory.

## Method

### Participants

Sixty-three individuals (♀ = 46, ♂ = 17) took part in this study ($\overline{{\text{M}}}$_age_ = 22.57 years; ${\text{SD}}$_age_ = 5.61 years). Participants were recruited from the Faculty of Psychology at the University Paris Descartes and from the cognitive sciences information platform (RISC/CNRS). They received either course credit or a small gift in exchange for their participation. All participants were required to have normal or corrected to normal vision, and to have never suffered from epilepsy. None of them reported having experienced symptoms of motion sickness due to virtual reality material prior to, during and after the experiment. Three participants were excluded from analyses for not following instructions. All participants signed a written informed consent form. Participants were randomly assigned to one of the two independent conditions (Active group = 31 participants, Passive group = 29 participants). For information about group matching, see Table 1. Sample size was determined using the G*Power software (version 3.1.9.7), with medium effect size (f^2^ = 0.15) and desired power = 0.80 as input parameters. As there was no Institutional Review Board (IRB) in our institution at the time of data collection (2017), no ethical approval was asked. In accordance with article L1122-1 of the French Public Health code and its subsequent amendments, ethical approval from an IRB is not mandatory for psychology research in France^47^. Accordingly, all procedures performed in the study were in accordance with the ethical standards of the institutional and national guidelines for psychology research, the French regulation for data protection (RGPD) and with the 1964 Helsinki Declaration and its later amendments or comparable ethical standards. The participants were informed that they could end the experiment at any time without having to justify their withdrawal, but none did.

**Table 1:** Mean and t.tests for group matching (SD in brackets).

	Demographic information & Habits Questionnaire											
	Age (years)	Height (cm)	Weight (kg)	Sport practice (hours per week)	Walk time (minutes per day)	Video-games time (hours per day)	Previous experience with HMD (from 0 to 1)	Motion sickness frequency (from 0 to 3)	Global estimated difficulty of the task (from 0 to 3)	Estimated cognitive difficulty (from 0 to 3)	Estimated physical difficulty (from 0 to 3)	Global satisfaction about performance (from 0 to 3)
Active condition	22.06 (4.47)	167.77 (8.01)	60.06 (9.69)	1.92 (2.26)	52.18 (32.28)	0.87 (0.91)	0.61 (0.49)	0.71 (0.78)	0.45 (0.62)	0.71 (0.64)	0.06 (0.36)	1.84 (0.73)
Passive condition	23.10 (6.62)	169.72 (7.95	65.22 (13.86)	2.05 (2.31)	66.38 (49.82)	1.03 (1.08)	0.55 (0.50)	0.9 (0.72)	0.48 (0.57)	0.97 (0.77)	0.10 (0.41)	2.21 (0.61)
t.test (p.value)	− 1.00 (0.32)	− 1.34 (0.18)	− 2.35 (0.02)	− 0.32 (0.75)	− 1.84 (0.07)	− 0.89 (0.37)	0.67 (0.50)	− 1.37 (0.17)	− 0.29 (0.77)	− 1.97 (0.05)	− 0.56 (0.58)	− 2.99 (0.003)

### Material

#### Virtual environment and virtual reality apparatus

**Figure 1:**
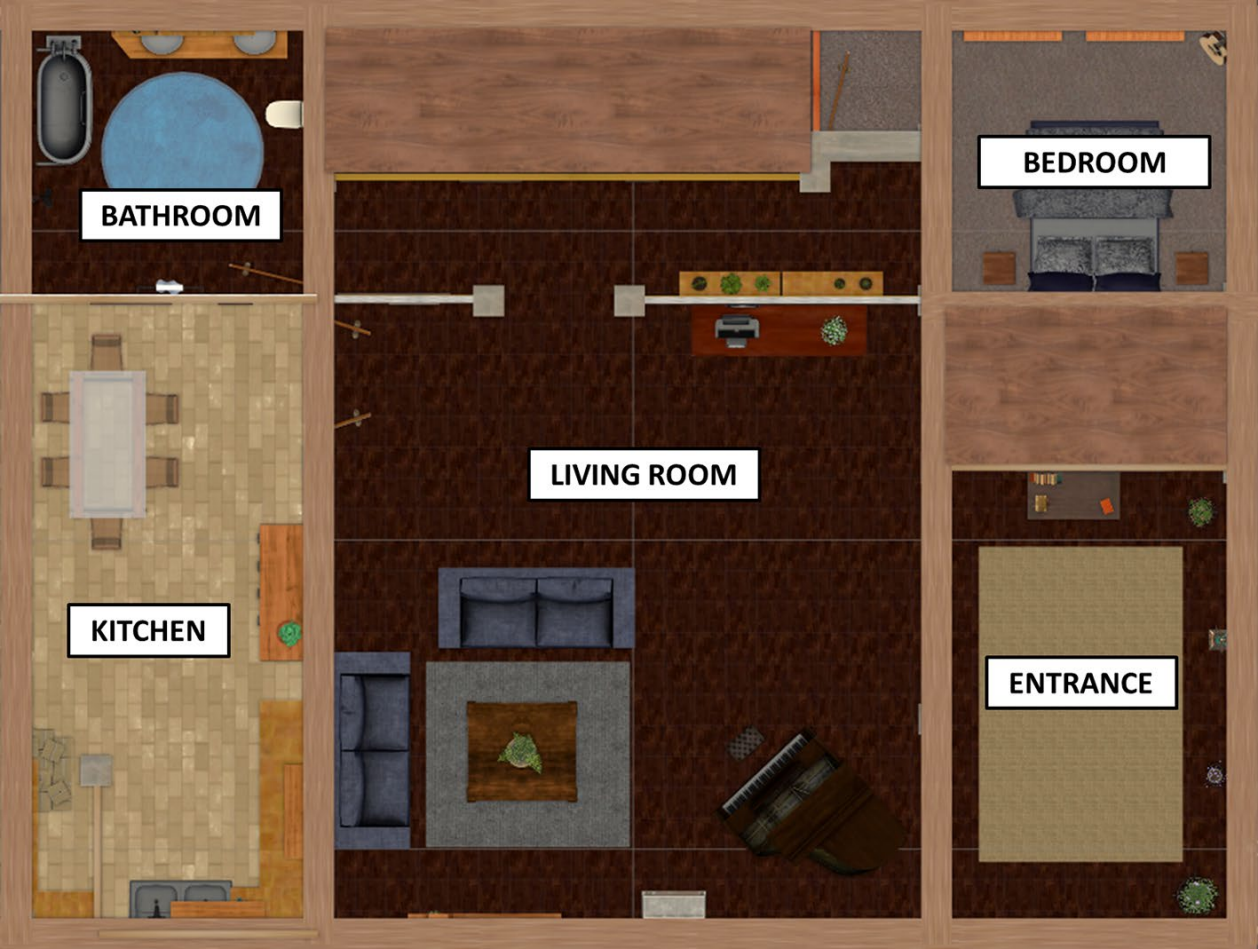
Top view of the virtual environment developed for this study, with the name of each part as mentioned during the training phase.

**Figure 2:**
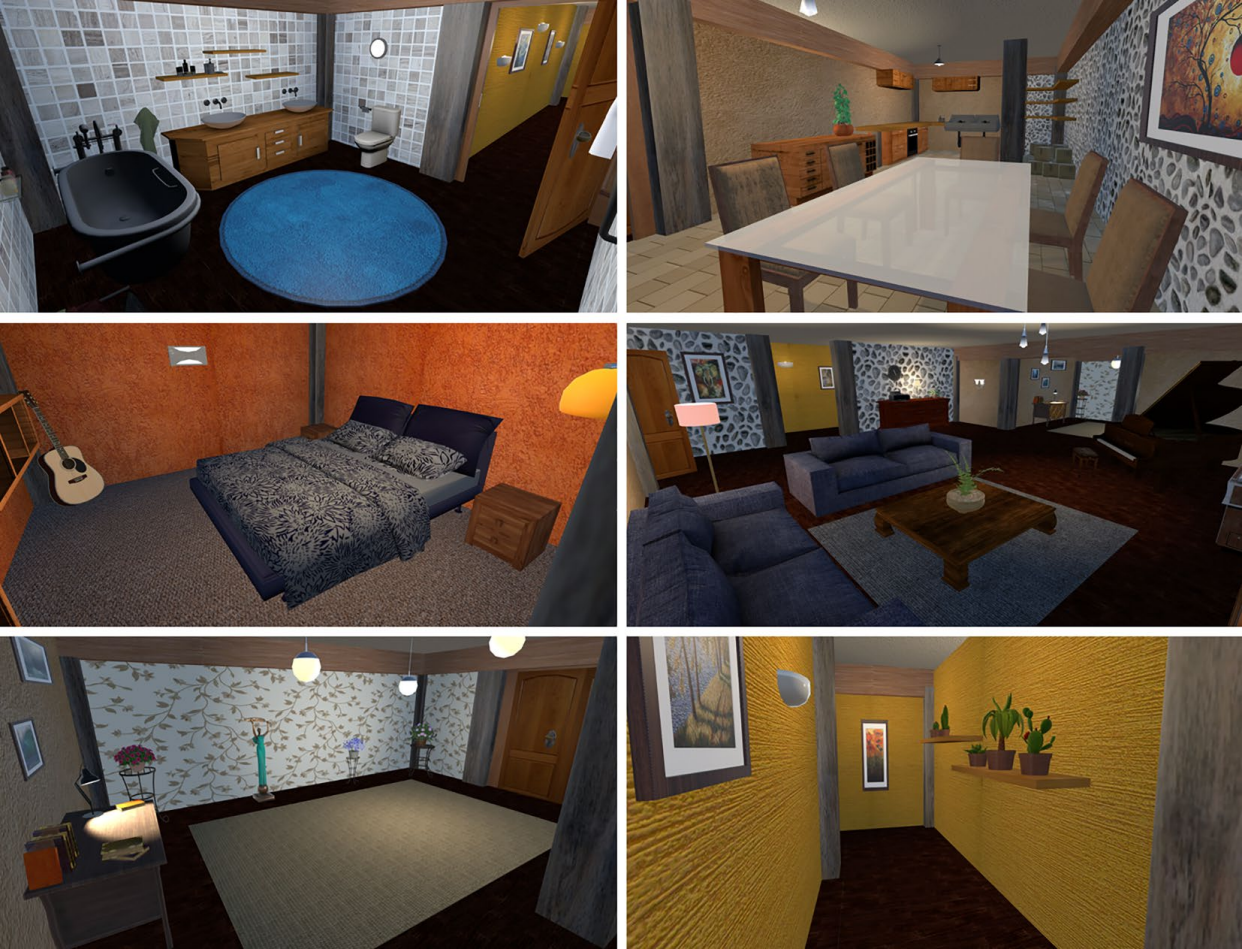
Photos of various locations within the virtual environment, taken from participants’ perspective**.** All the objects used to decorate the virtual scene were static and were positioned so as not to interfere with the movement of the participants or the visibility of the items during the learning phase.

A realistically scaled virtual interior house (ground surface = approximately 30 × 30 m) was created for the needs of this study using Unity Software v.2017.3.0f3 (https://unity3d.com/). Five different rooms and one corridor were designed using easily recognizable furniture and textures to avoid participants confusing them (see Fig. 1 for a map and Fig. 2 for examples of visual scenes from the participant’s perspective). The environment was designed to be used with an HTC Vive head-mounted display and controllers and navigation was possible by actual walking for short distances (in a 3 × 3 m area) and using the Steam VR teleport plugin for longer distances. Teleporting consisted in pressing a button on a controller to make a spatial target sight appear, pointing on a visible location with this sight and then releasing the button to instantly teleport to the aimed location. The external boundaries of the walking area were signaled by a holographic grid appearing only when they are about to be crossed.

#### Images

**Figure 3:**
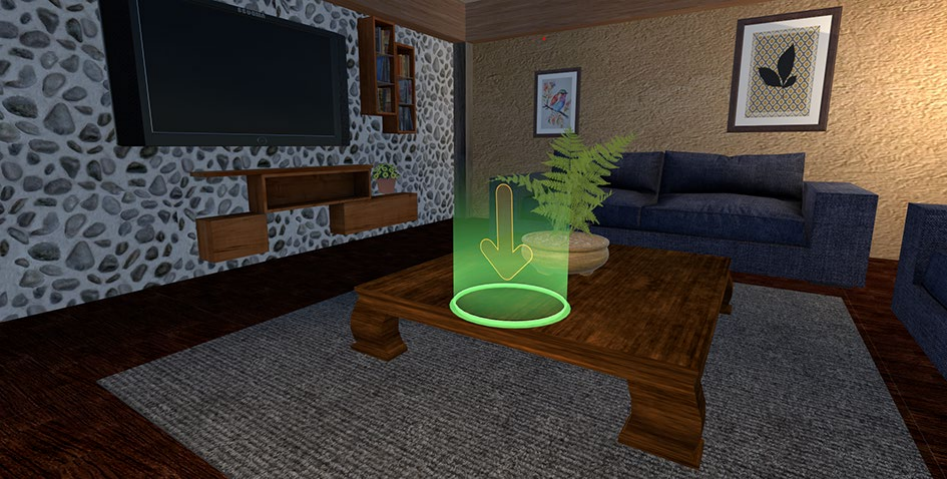
Example of a spatial target position in the virtual environment, as indicated for both the passive and active participants. As participants approached the spatial target position symbolized by the green and orange hologram, it was replaced by the image of the object (passive group) or it disappeared and the image of the object appeared in front of them (around 40 cm from participants’ head at eye level) so that they could grab it and drop it using a downward arm motion (active group).

**Figure 4:**
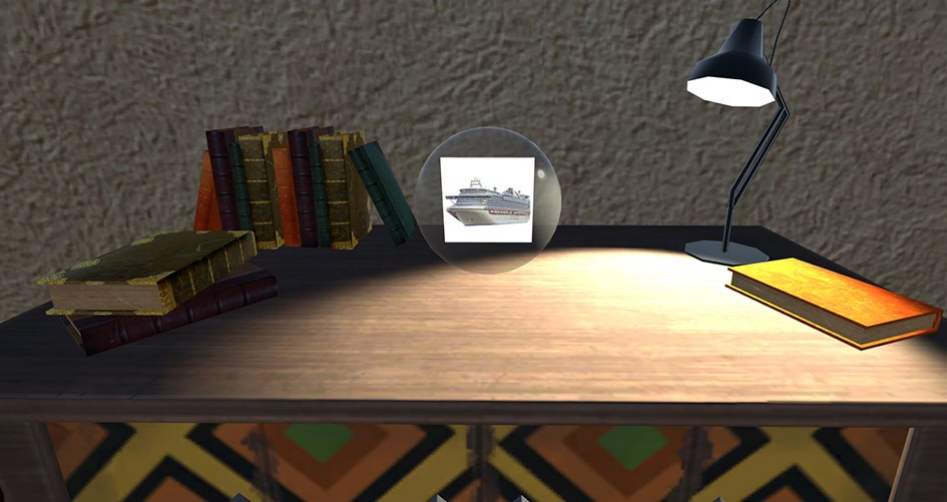
Example of an object positioned during the learning phase from the participant's point of view, after the participant has approached and the spatial target disappeared and replaced by the item (passive condition), or after it has been dropped in the position that was designated by the spatial target (active condition).

The stimuli used for this study were high resolution (2000 × 2000 pixels) color pictures of 8 highly manipulable objects and 8 less manipulable objects presented on a white background. Highly-manipulable objects were frequently handled objects such as tools. Every highly manipulable object chosen for this study had a grip implying the use of power grabbing (e.g., hammer, watering can). Less manipulable objects were infrequently handled objects of various sizes (e.g., boat, lighthouse, dragonfly…). All objects were chosen from a standardized database^48^ using only more than 90% grasp agreement objects. From now on, we will refer to the two categories of objects used in the study as manipulable (for highly manipulable) and non-manipulable (for less manipulable) items for simplicity's sake (although the degree of manipulability is expressed on a continuous axis according to the database). Objective lexical frequency was controlled using the LEXIQUE online database (http://www.lexique.org/^49^) to ensure that the concepts associated with the images are all equally familiar to participants and that they do not differ across manipulability in this regard (t_(16)_ = − 0.49, *p* = 0.62). All objects were presented as images embedded in 30 cm diameter 3D spheres (See Fig. 4) so that the interactive shape of the item was the same for manipulable and non-manipulable objects. That way, we avoided representing the objects as miniature 3D models, which would have turned manipulable some objects that cannot be handled in reality (for instance, the miniature model of a boat can be manipulated, whereas the usual concept of a boat is non-manipulable). All images in spheres were programmed to automatically face the position of the HMD in order to make sure that they were all properly seen by participants. The list of manipulable items included images of the following words: “hammer” (*marteau*), “axe” (*hache*), “hairdryer” (*sèche-cheuveux*), “iron” (*fer à repasser*), “frying pan” (*poële*), “drill” (*perceuse*), “flashlight” (*lampe-torche*) and “watering can” (*arrosoir*). The list of non-manipulable items included images of the following words: “lighthouse” (*phare*), “zebra” (*zèbre*), “water lily” (*nénuphar*), “fountain” (*fontaine*), “boat” (*bateau*), “garden gnome” (*nain de jardin*), “dragonfly” (*libellule*) and “barrel” (*tonneau*) (Figs. 3, 4).

#### Spatial position retrieval task

A pencil-and-paper position retrieval task was used to assess participants’ memory for the objects’ location and to collect information about metric properties of the spatial representation derived from item position learning. This consisted of a sheet of paper (A4 format) with a black rectangle (15 × 11 cm) printed in the center for participants to draw a map of the virtual environment. The names of the pictures were written around the rectangle (in a random order and arrangement), to ensure that participants were only tested on spatial positioning and not on retrieving images.

#### Questionnaire

A questionnaire was created and used to gather information about participants’ profiles and their experience during the experiment. It was composed of eight items on a 4-point scale (from “Not at all/Never” to “Absolutely/Always”) and four items allowing open numeric responses (e.g., “*How many hours a week do you spend on sports/exercising*”). The aim was to collect individual information about the frequency of physical activities/video-games usage, and information about the task performed during the experiment: motion-sickness experience, cognitive and physical perceived difficulty, and global satisfaction about the performance (see Table 1).

### Procedure and design

**Figure 5:**
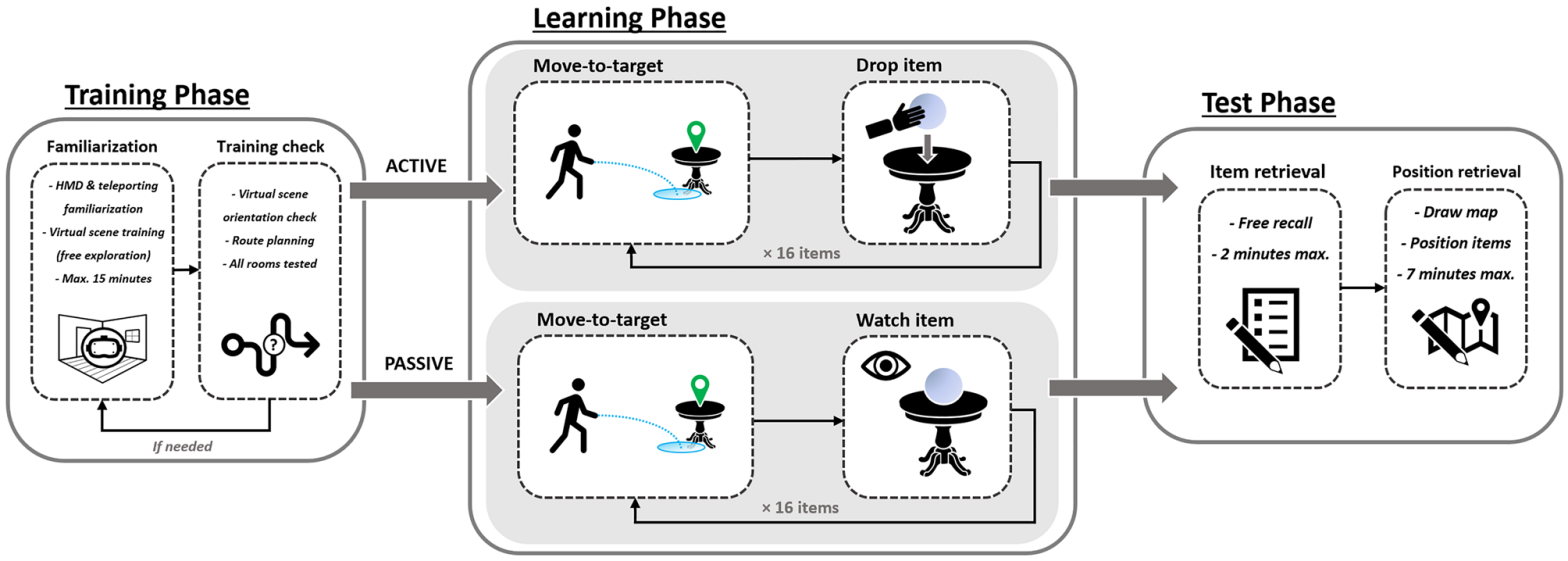
Summary of the experimental flow. Participants begin the experiment with a training phase including familiarization, then perform the learning phase and are finally tested on item and position retrieval.

**Figure 6:**
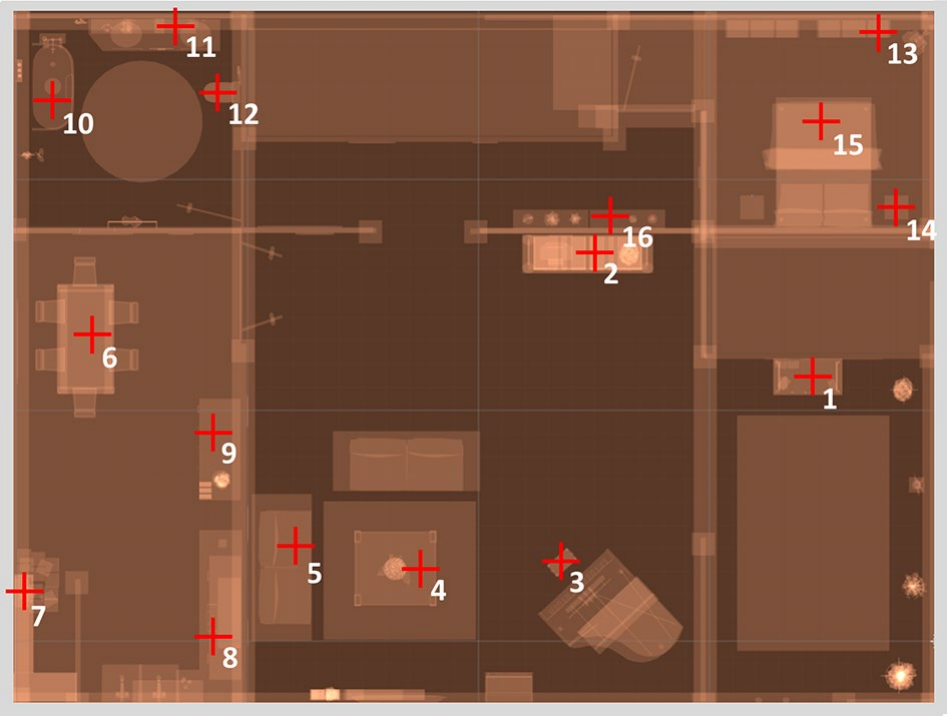
Positions and order of presentation of the items during the learning phase indicated here by their number.

**Figure 7:**
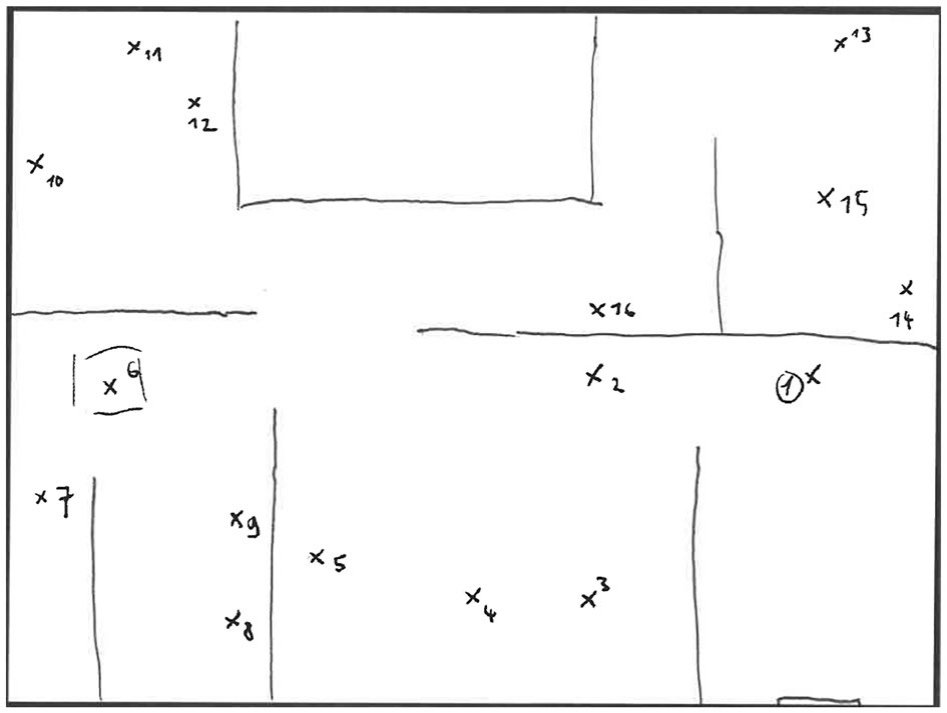
Example of a map drawn by a participant (passive condition) during the item position retrieval task. Instructions were to draw at least walls and to position items on the map.

Each participant was randomly assigned to one of the two independent groups (Active group or Passive group) and was tested individually for approximately 30 min. After they had been asked to communicate their socio-demographic information, participants were equipped with the head-mounted display and the controllers. They then started the experiment by a training phase during which they had to train teleporting through the environment, as well as learn the general layout of the rooms (free exploration by navigation). When participants began to feel familiar with the apparatus and the environment, the experimenter asked them to perform simple route planning trials to verify that they knew the locations of the different rooms (e.g., “go to the bedroom”, “go to the television in the living room”; five different locations were tested). If participants were unable to orient themselves at this point, they were given extra navigation time and were tested again. The learning phase then begins. Instructions were given orally. If participants were previously assigned to the active group, the instructions were: “You will now see a spatial target appear somewhere in the environment. The spatial target is a hologram pointing to a specific location, that you can see through walls. You will have to move towards this spatial target. Once you are about a meter away from the target hologram, an item will appear in front of your head. This item will be an image floating in a glass bubble. You will have to take the item in your hand by pushing the trigger of the controller, and to put it down at the exact location of the spatial target. When an item is positioned, the spatial target disappears and then reappears in another location, with another item. Your task is to memorize all the images/items with their respective locations, because you will have to retrieve them and their positions on a map later”. Participants in the passive group underwent the same procedure and instructions, with the exception that when they arrived within one meter of a target-hologram, the item appeared directly at the target position. Unlike in the active group, the passive participants therefore did not have to perform voluntary reaching movements and thus have no agency over the positioning action. Active and passive participants underwent the same navigation protocol, using teleportation for long inter-items distances and then adjusting their position to approach closer to the item by walking a few steps. Once participants understood the task, the experimenter started the protocol by triggering the first spatial target. No time limit was imposed for the learning phase. Active and passive participants both saw all sixteen items and positions, half of the items being manipulable objects and the other half being non-manipulable objects. Spatial targets and associated items appeared one at the time, and participants were asked not to go back to previous items. Spatial target locations and order of locations were always the same (see Fig. 6 for a map of these locations with their order of appearance order; see Fig. 3 for an example of the spatial target as seen by participants), meaning that navigation trajectories were always the same for all participants and all items were seen from the same viewpoint to control for visual experience across participants. Assigning the items to positions was pseudo-randomized: three order lists were created to control for serial effects across manipulable and nonmanipulable items, but also to make sure that no clusters of several items of the same type were randomly created and placed contiguously (participants were randomly assigned to one of the lists for the learning phase). Objects were never assigned to locations that were semantically congruent with their use or attributes. Once the sixteen items had been positioned, the experimenter announced the end of the learning phase and removed the apparatus from participants’ head. After that, participants were comfortably seated on a table and the test phase began. They were first asked to write down as many items as possible in a free recall task. The time limit was 2 min and they were free to recall items in any order (only items, not positions). They were then presented with the item position retrieval task, in which the instructions were to draw a simple map of the house (only walls were required) in the central rectangle in order to position each item on the map at the exact location it occupied during the learning phase (see Fig. 7 for an example). All the items had to be positioned to complete this task. The time limit was 7 min. Lastly, participants completed the questionnaire before receiving a debriefing by the experimenter, describing the goals of the study (Figs. 5, 6, 7).

## Results

### Measures

Item spatial position retrieval task performances were analyzed using the GMDA software^50^ to extract spatial coordinates from participants’ sketch maps and perform geometric analyses on them. Using this software, we obtained three different measures: (1) *distance accuracy* (2) *correlation coefficient* (*r*) (3) *scale*. The *distance accuracy* score is computed from pairwise landmark comparisons, which summarizes two by two inter-item distance magnitude errors (compared to the reference map) under the form of absolute distance error ratios. It provides an indicator of global pairwise distance accuracy without taking into account directionality. This score ranges from 0 to 1, higher scores meaning higher pairwise distance fidelity with the reference layout.

The GMDA software is also able to compare sketch map coordinates to actual reference coordinates using bidimensional regression^51^. This method is particularly relevant to study how cognitive maps are distorted with respect to topographic reference layouts^52^. Bidimensional regression yields different measures among which we chose two dependent variables to analyze: the *r* and the *scale* measures. The *r* score is equivalent to a Bravais-Pearson correlation coefficient adapted for bidimensional regression. It reflects the spatial fit between sketch maps and actual reference item layout and can be interpreted as a global configurational similarity measure ranging from 0 (no relation between the sketch map and the reference coordinates) to 1 (perfect match between the positions of the two sets of coordinates). This index is not sensitive to translational, rotational or scaling errors. An *r* score close to zero would indicate that the coordinates have been recalled almost at random and would invalidate any interpretation concerning the metric properties of the representation. The second measure, which is the *scale* index, accounts for the global directional scaling error of all Euclidean distances. Values below one reflect global scaling compression for the sketch map compared to the reference layout, while values above one indicate global scaling expansion. The *scale* and *r* measures obtained from the bidimensional regression analysis are orthogonal, which means that a highly expanded or compressed representation can still correlate well with the reference map if most proportions are preserved. *Distance accuracy* and *scale* extract different information, the former summarizing pairwise absolute distance errors between items, and the latter describing cognitive maps’ directional expansion or compression as a whole. Bias-related pairwise measures such as computed by the GMDA software are not correlated with bidimensional regression *scale* because distance ratios are better suited for representing inter-landmark distances estimations from memory^50^. We used these two measures to better characterize metric errors: if *distance accuracy* is low but *scale* is close to 1, this indicates that distance errors are large but their direction is random. Conversely, if *distance accuracy* errors are combined with a lower *scale* score, this would indicate that metric errors are systematically biased toward compression.

### Statistical analyses

All analyses were conducted using R software version 3.4.0^53^. Mixed linear regression models were conducted using the lme4 package (version 1.1-8^54^). All mixed models used motor activity (comparing active to passive condition) and object manipulability (Comparing manipulable to non-manipulable items) as fixed effects factors, as well as the interaction between these two predictors. Random intercepts were entered for the participants in order to account for inter-individual variability. We also entered the free recall task scores as a random effect with random intercepts to account for the fact that participants who memorized items better were more likely to better retrieve their spatial positions. Similarly, participants who can't remember items are more likely to position them randomly on the map. Using free recall scores as a random effect thus allows for a better distinction between specific spatial performance and (non-spatial) memory performance. This analysis protocol was strictly repeated for three different measures: the correlation coefficient (*r*), *distance accuracy* and *scale* indicators computed with the GMDA software from the item spatial position retrieval task performances on maps (see the “Measures” part). The homogeneity of variances between the distributions of the conditions was assessed using Levene’s test. All scores were transformed using Tukey’s Ladder of Power Transformations^55^ to correct for skewness and non-normality of the distributions. Five participants were excluded for recalling less than six items and showing abnormally low correlation scores (r < 0.3). Outlier detection was performed using Cook’s Distance^56^ for each model, using a classical cutoff value of *Di* > *4/n* (*Di* = computed Cook’s distance) to remove abnormally deviant values^57^ before computing the final model. This entire process resulted in the removal of approximately 9% of the observations for each measure.

### Correlation coefficient

**Figure 8:**
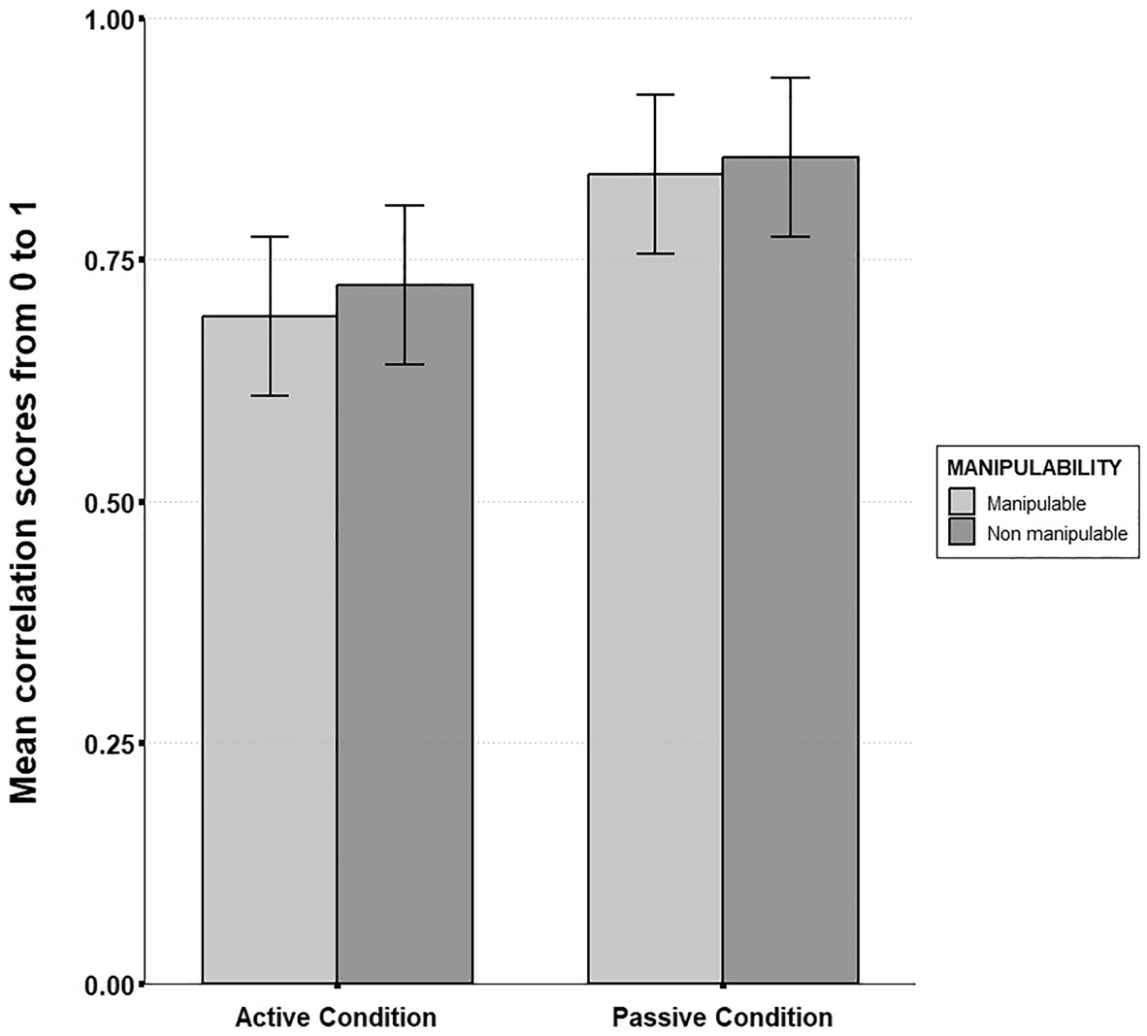
Mean correlation coefficient scores from bidimensional regression as a function of motor activity and object manipulability (Error bars are standard deviations). A significant main effect of motor activity was observed (p. < 0.01). No significant main effect of object manipulability was observed. No significant interaction effect was observed.

A significant coefficient was found for the correlation coefficient score, revealing a main effect of motor activity on the global configurational correlation between participants’ sketch maps and reference layout (β = 0.179, SE = 0.07, t = 2.73, p = 0.007). Actively manipulating items during spatial learning was associated with lower correlation scores ($\overline{M }$_active_ = 0.74, SD = 0.22; $\overline{M }$_passive_ = 0.85, SD = 0.18). Item manipulability did not significantly predict correlation scores (β = 0.055, SE = 0.04, t = 1.31, p = 0.203). No interaction effect was found between motor activity condition and item manipulability (β = − 0.025, SE = 0.04, t = − 0.523, p = 0.608; see Fig. 7) (Fig. 8).

### Distance accuracy

**Figure 9:**
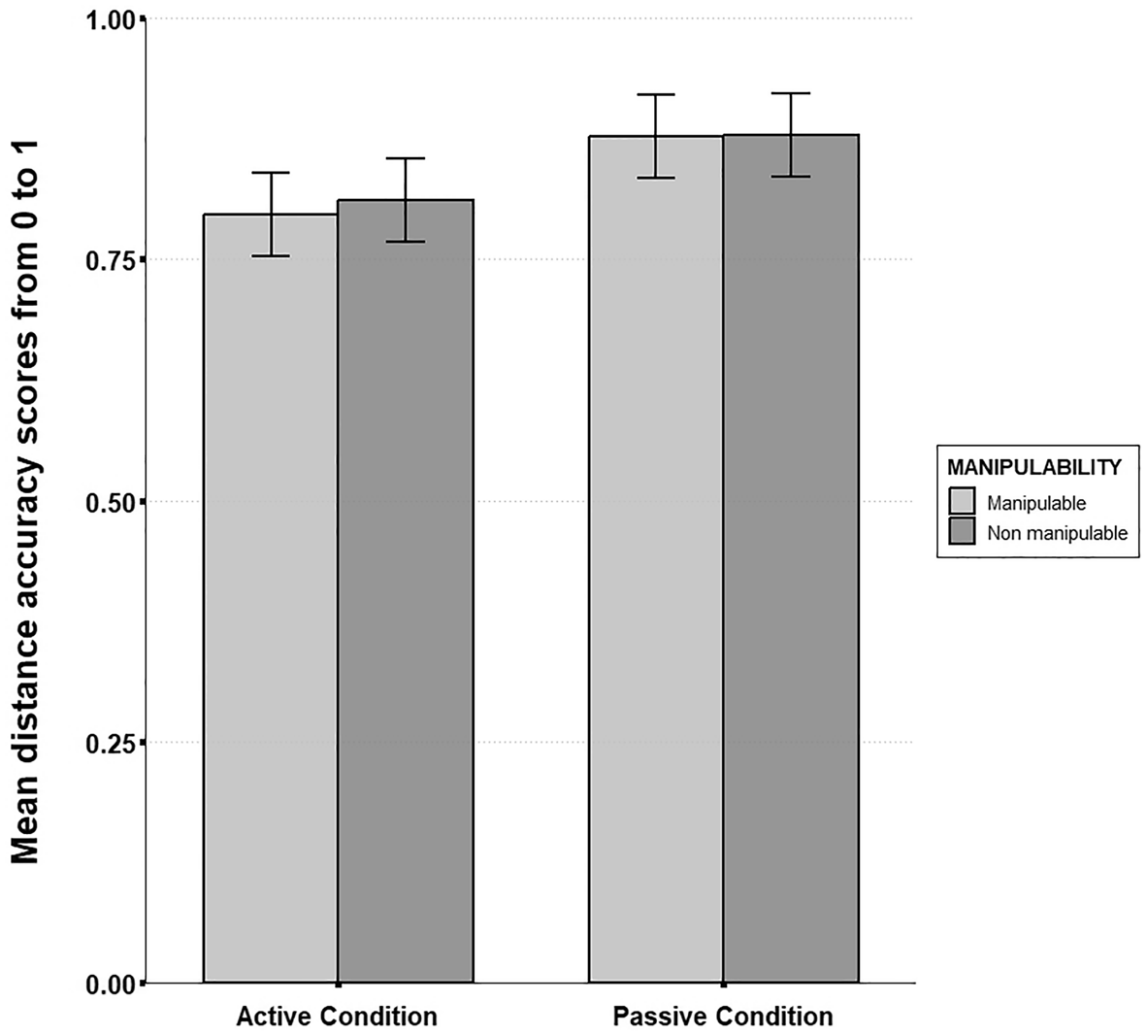
Mean distance accuracy scores from the GMDA software as a function of motor activity and object manipulability (Error bars are standard deviations). A significant main effect of motor activity was observed (p. < 0.05). No significant main effect of object manipulability was observed. No significant interaction effect was observed.

Motor activity significantly predicted distance accuracy (β = 0.04, SE = 0.01, t = 2.31, p = 0.023). Participants in the active condition made more absolute inter-landmark distance errors than participants in the passive condition ($\overline{M }$_active_ = 0.8, SD = 0.07; $\overline{M }$_passive_ = 0.88, SD = 0.07). However, manipulability did not affect absolute distance accuracy (β = 0.018, SE = 0.01, t = 1.38, p = 0.18) and no interaction effect was observed between motor activity condition and item manipulability (β = 0.012, SE = 0.01, t = 0.74, p = 0.46; see Fig. 8) (Fig. 9).

### Scale

**Figure 10:**
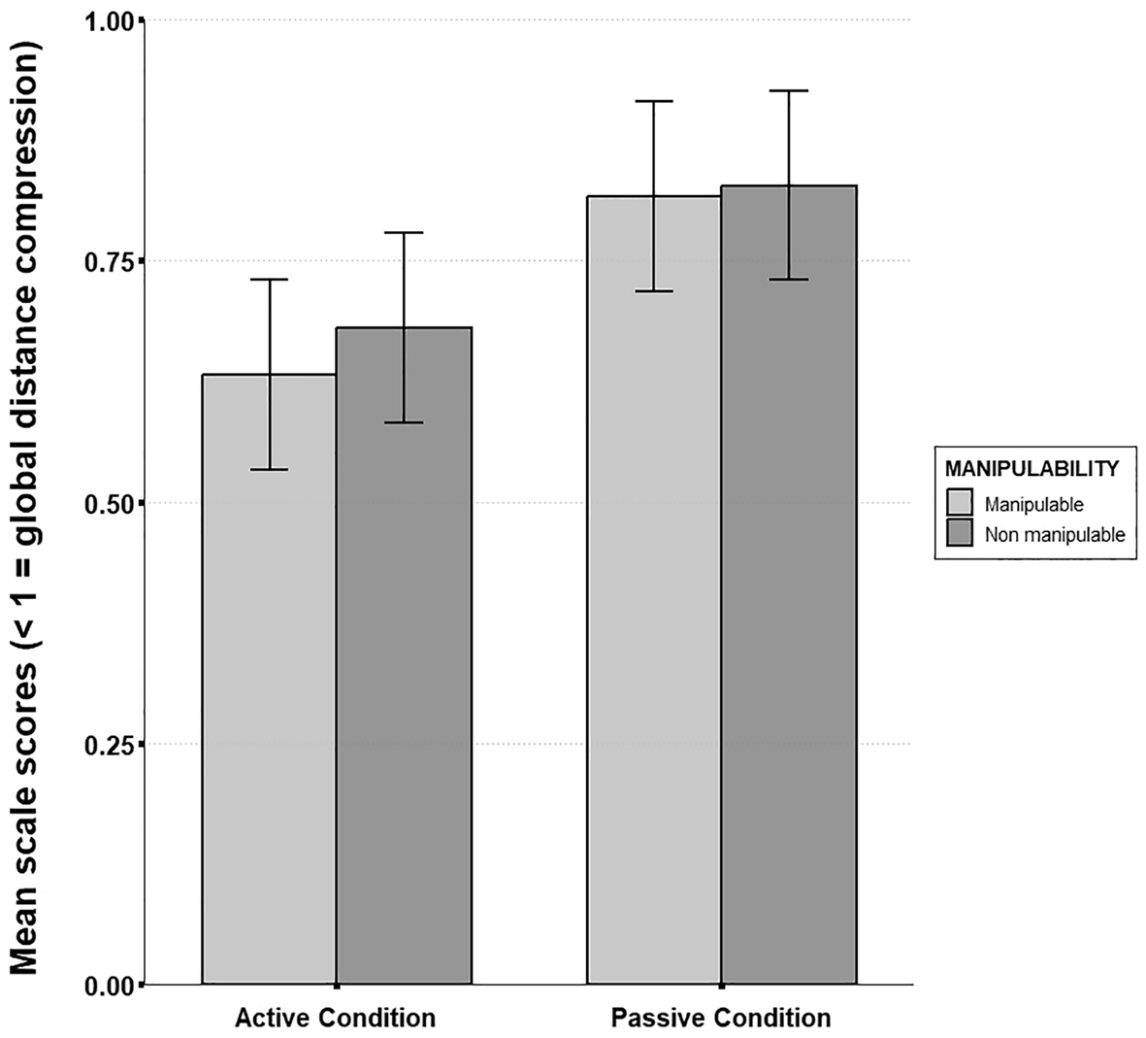
Mean scale compression in the items position retrieval task as a function of motor activity and object manipulability (Error bars are standard deviations). Both motor activity (p. < 0.01) and object manipulability (p. < 0.05) yielded significant main effects. No significant interaction effect was observed.

A significant coefficient was found for motor activity regarding scale scores (β = 0.229, SE = 0.07, t = 3.2, p = 0.001). While all participants tended to compress the global scale of their representation compared to the reference layout, this compression was higher for the active group than for the passive group ($\overline{M }$_active_ = 0.66, SD = 0.24; $\overline{M }$_passive_ = 0.82, SD = 0.21). Item manipulability also yielded a significant effect for scale scores (β = 0.122, SE = 0.05, t = 2.3, p = 0.03), indicating that manipulable items led to more compression than non-manipulable ones ($\overline{M }$_manipulable_ = 0.72, SD = 0.26; $\overline{M }$_non-manipulable_ = 0.75, SD = 0.22). No interaction effect was found between item manipulability and motor activity condition (β = − 0.071, SE = 0.07, t = − 1.1, p = 0.3; see Fig. 9) (Fig. 10).

## Discussion

The present study investigated the effect of motor reaching activity and object manipulability on the metric properties of large-scale spatial representations. It aimed to show that motor interactions in peripersonal space could influence the estimation of metric properties in the spatial representation retrieved from memory. To do so, we asked participants to learn associations of items and spatial positions in immersive virtual reality. In an active condition, participants had to physically extend the arm to reach the items and drop them at specific locations. In a passive condition, the items appeared in position at the spatial target location, and participants did not have to physically interact with the items. Items were images of manipulable and non-manipulable objects. Participants were tested on a sketch map drawing with an item positioning task. Results showed that actively interacting with objects distorted metric properties in spatial memory: the shape of the retrieved spatial layouts was less correlated with the actual layout, and all pairwise distances between landmarks were less accurate. More precisely, we observed that active participants showed greater global compression of the scale of their representation than passive participants, which suggests that actively manipulating items biases distance memory in a specific direction, consistent with previous work on reaching and distance estimation^33,38^. These findings replicate the ones obtained by Thomas et al.^44^ and Clement et al.^45^, while using a more complex large-scale environment and taking advantage of bidimensional regression to provide quantitative descriptions of different metric errors from memory. Furthermore, we proposed motor simulation as one of the mechanisms contributing to interaction-related metric errors, and operationalized it using item manipulability. While the expected interaction effect between item manipulability and motor reaching activity was not observed, we still found that manipulable items were associated with more global compression than non-manipulable items.

First of all, our results showed that manipulable items were less well recalled than non-manipulable items during the free recall task. This unexpected result could be explained by the fact that manipulable items belong to more similar and less diverse semantic categories than non-manipulable ones (e.g. “tools”, “cooking utensils”…) and are more prone to be encountered in the present context (a house), thus reducing their distinctiveness in memory^58^. However, we also observed that manipulable items were associated with more distance underestimation than non-manipulable items during spatial positions retrieval. This is in line with our hypothesis stating that manipulable items are more prone to generate distance biases from memory than non-manipulable items. However, we predicted that this effect would interact with motor activity. While descriptive statistics suggest that the effect increased for the active group (see Fig. 9), inferential analyses did not reveal any significant interaction effect. We are thus not able to conclude on our initial hypothesis that action-related processes primed by manipulable concepts^20^ are strengthened by active manipulation during spatial encoding. This implies more complex interactions between different sources of body-related information than mere additive effects, and should be further investigated in future studies.

Regarding the active condition effect, the global underestimation of distances in memory observed here is in line with early studies indicating that distances in memory could be retrieved in relation to the body: for example, objects that were close to the protagonist during spatial learning were processed faster on a lexical decision task than distant objects^42,43^, suggesting that object accessibility is encoded in memory in an action-directed way. Generally, it has been shown that alignment of the object with functional axes of the body is a determining factor in the ease with which positions are recalled from memory, thus arguing in favor of a “spatial framework” where action possibilities and motor asymmetries drive spatial memory^25^. Our results suggest that actively manipulating items during spatial learning can cause distortions of the metric properties of the spatial representation, because of the existence of a body-related spatial framework that emphasizes the functional value of action in memory^22^. Consequently, the present results constitute a new element in favor of a constructivist and embodied view of spatial representations, such as the “spatial collages” metaphor of Barbara Tversky. This view postulates that spatial representations are thematic overlays of multimedia information about the structure of the environment^26,27^. Such a vision of spatial representations predicts that the information is systematically distorted, in particular because of the inclusion of non-spatial information such as motivations or action capabilities^59^. Physical reaching actions during learning thus lead to the encoding and retrieval of non-isomorphic spatial representations in which distances are locally compressed. This compression would be due to the integration of information about the outcomes of action possibilities within the arm reach space. This compression effect could be due either to a transfer of perceptually rescaled distances into the spatial representation, or to a retrieval of action possibilities information from memory that distorts the metric properties during the retrieval phase, and the exact stage at which motor resources are used and may cause metric biases should be investigated. Future work should also address the notion of agency in active learning, since "active" participants were also active insofar as they carried out participatory learning, in which motor activity was mediated (arm extension, with a symbolic grabbing action).

However, this action-related bias on participants’ memory of the metric properties could also be explained by the fact that the active condition was more cognitively demanding than the passive condition in our experiment. This is substantiated by the difference in correlation scores between active and passive condition: active participants’ sketch maps are less similar to the actual layout than passive participants’ sketch maps. Although it is possible that active learning was more difficult than passive learning because of the inclusion of additional steps (grasping and dropping items), answers given by participants to the questionnaire confirmed that active participants did not find the global and physical task significantly more difficult than passive participants. The active group even reported significantly lower scores for the estimated cognitive difficulty of the task compared to the passive group, which suggests that the hypothesis of increased mental load associated with active manipulation can be ruled out (see Table 1). Additionally, difficulty disparity between conditions cannot account for the specific direction of distance biases from memory: correlation scores and scaling errors are orthogonal when using bidirectional regression^52^. If the effect of the active condition was only due to the difficulty of the task, then we would have observed only an increase in absolute distance errors, with no obvious directionality. However, we found that active manipulation had a specific directional effect in the form of distance compression, which has been associated with action capability effects in previous studies^33,60^.

As noted in the introduction, action capability effects also appear in connection with participant characteristics such as arm length, which influences the estimates of distances contained in the peripersonal space according to perceived arm reach^61^. This suggests that immediate distances perception is shaped by morphology^62^ and individual arm length could have had an effect on our results. Although we did not measure this parameter, we argue that in our case such individual characteristics is unlikely to significantly influence our results because it should only affect the magnitude of the compression induced by the manipulation rather than alter its direction, given that all objects were within the arm reach perimeter located in the peripersonal space. Furthermore, using linear mixed models which calculated random intercepts per subject aims to account for the inherent individual variability of participants. If action possibilities offered during spatial learning distort the representation in memory, then other action-related effects should also impact this scaling mechanism. For example, the use of tools that extend the arm reach range towards objects leads to underestimating immediate distances^63^, and should thus increase the amplitude of the compression bias observed in our protocol if this factor was to be manipulated for future studies. More importantly, we know that visual perception of immediate distances is affected by action constraints (for a review see^64^). In particular, it has been shown that an object that is more difficult to reach and grasp due to the presence of obstacles^65^ or because of the orientation of its handle^37^ is conversely perceived as farther. If perceived action possibilities are actually encoded in the spatial representation and implicitly shape its metric properties, then these specific examples of action-related biases would lead to an expansion of its global scale. Future studies should thus focus on the transfer of perceptual biases that can distort spatial representation in directions other than scale compression.

As a whole, the observed main effects of active motor learning and item manipulability are in line with embodied views of memory. According to the “Act-In” model of Versace et al.^23^, memory traces are multimodal in that they contain all sensory information gathered from experience, distributed across multiple neuronal systems. Therefore, the emergence of specific knowledge during memory retrieval may arise from the interactive activation of sensory experiences, leading to the reenactment of these components through the simulation mechanism^23^. As spatial knowledge is also built from a categorization process of possible interactions with the environment^66^, it seems possible that retrieving the positions of items in our study involved the simulation of sensory components experienced during learning, especially motor components of physically grabbing objects for the active group. According to the theory of perceptual symbol systems of Barsalou^5^, this also appears to be the case for manipulable concepts, whose salient features automatically involve motor simulation of hand and arm movements, thus explaining the arm reaching bias on retrieved distances observed for these items in the active group.

Taken together, these results are in line with embodied theories of memory and goal-directed views on spatial cognition: they point out that physically interacting with objects can distort the metric properties of the global spatial representation of the environment. This can be considered as another argument that spatial representations in memory integrate multimodal information derived from experience^26,27^. Moreover, it shows that classical effects of arm reaching observed for physical spatial perception^33,37,38^ can also be observed regarding mental spaces in memory: physically interacting with objects may lead to some localized narrowing of scales, thus advocating for a non-isotropic nature of spatial representations centered around the body, as a primary reference point.

### Supplementary Information

Supplementary Information.

## Data Availability

All data generated or analysed during this study are included in this published article and Supplementary information files.
